# Usability of simplified audiometry and electrocardiogram during treatment of drug-resistant tuberculosis in Mozambique: a qualitative study

**DOI:** 10.1186/s44263-024-00039-4

**Published:** 2024-02-14

**Authors:** Pedroso Nhassengo, Américo Zandamela, Celina Nhamuave, Sheyla Rodrigues Cassy, Rogério Chiau, Cláudia Mutaquiha, Pereira Zindoga, Ivan Manhiça, Celso Khosa, James Cowan

**Affiliations:** 1 Instituto Nacional de Saúde, Marracuene, Moçambique; 2 Departamento de Matemática e Informática, Faculdade de Ciências, Universidade Eduardo Mondlane, Maputo, Moçambique; 3 Departamento de Matemática, Faculdade de Ciências e Tecnologia, Universidade Nova de Lisboa, Lisboa, Portugal; 4 Health Alliance International, Maputo, Moçambique; 5 Programa Nacional de Controlo da Tuberculose, Ministério da Saúde, Maputo, Moçambique

**Keywords:** Usability, Electrocardiogram, Audiometry, Drug-resistant tuberculosis, Mozambique

## Abstract

**Background:**

In 2021, there were approximately 450,000 cases of drug-resistant tuberculosis (DR-TB) worldwide. The treatment of DR-TB historically included expensive and toxic injectable drugs leading to adverse effects including ototoxicity and Electrocardiogram (ECG) abnormalities. This study described the perspectives of healthcare providers and people with DR-TB on the usability of simplified audiometry and ECG for monitoring treatment adverse effects.

**Methods:**

A qualitative study was conducted in December 2019 across four provinces in Mozambique, namely Maputo, Gaza, Zambézia, and Nampula. Sixteen outpatient primary care health facilities equipped with simplified Audiometry and/or ECG devices (specifically, SHOEBOX Audiometer® and/or SmartHeart Pro ECG®) installed for at least 6 months before the study initiation were selected. The data was collected using in-depth interviews (IDI) and Focus Group Discussions (FGD) techniques. Interviews were audio-recorded, transcribed verbatim in Portuguese, coded, and analyzed using Nvivo 12 software®. We generated two themes and fit our results into a conceptual framework consisting of three domains in the implementation of technological innovations in health.

**Results:**

A total of 16 healthcare providers and 91 people undergoing treatment for DR-TB were enrolled in the study. Most people with DR-TB had experienced audiometry testing and demonstrated a good understanding of the assessments. Conversely, while most healthcare providers demonstrated robust knowledge of the importance of both audiometry and ECG assessments, they were not confident in managing ECG devices and interpreting the results.

**Conclusions:**

While healthcare providers demonstrated a consolidated understanding of the importance of audiometry, the limited number of devices and lack of training were constraints, impeding optimal usage and service delivery.

**Supplementary Information:**

The online version contains supplementary material available at 10.1186/s44263-024-00039-4.

## Background

In many low- and middle-income countries (LMICs), drug resistant tuberculosis (DR-TB) remains a significant public health challenge. The World Health Organization (WHO) estimated 450,000 cases of DR-TB worldwide in 2021 [1]. In Mozambique, 3.7% of the notified 97,000 people, had DR-TB [2]. The Mozambican Ministry of Health recently updated the DR-TB guideline, aligning it with the WHO reference guidelines [1, 3]. The updated guideline incorporated a shift from injectable to oral regimens, the adoption of shorter treatment regimens, and expanded the use of delamanid (DLM) and bedaquiline (BDQ) as part of individualized treatment regimens [1].

People with DR-TB are at risk of suffering from serious adverse effects, such as irreversible hearing loss due to aminoglycoside toxicity (an injectable antibiotic that was until recently a core component of the recommended DR-TB treatment regimen) [4–6] and electrocardiogram (ECG) abnormalities from BDQ [7], DLM, clofazimine, moxifloxacin and other medications posing a risk of sudden death for people under treatment. Previous studies underscore the prevalence of irreversible hearing loss among people with DR-TB treated with aminoglycosides [8, 9].

Currently, all people with DR-TB in Mozambique who are eligible for individualized DLM or BDQ regimens have their files reviewed by the National Clinical DR-TB Committee, which meets weekly [3, 10]. As of May 2018, the Mozambican National TB Program (NTP), in collaboration with Health Alliance International (HAI), a global health non-governmental organization, decided to pilot and implement an innovative integrated tablet platform at 50 high-volume DR-TB health facilities throughout the country. The tablet platform provided a comprehensive solution by integrating three core functionalities: (1) Audiometers ShoeBox®/App to offer simplified audiometry screening; (2) 12-lead ECG SmartHeart® Pro testing (implemented with a separate Bluetooth-enabled ECG device) with algorithms that calculated the QTcF interval, and (3) Zoom application to participate in weekly DR-TB case discussion meetings. People with DR-TB were assessed using the simplified devices, namely the Audiometer ShoeBox® and ECG SmartHeart® Pro. The assessments occurred at treatment initiation and continued monthly during the use of injectable drugs. While this is a significant step towards offering better monitoring of treatment adverse effects, the implementation and expansion of DR-TB therapies in LMICs can be limited by the lack of accessible and simplified drug toxicity monitoring platforms to identify people eligible for individualized regimens [1]. Studies on TB treatment adverse effects using simplified audiometers and ECGs reinforce the need for the allocation of similar devices at the primary care health facility [11]. However, the usability of these simplified devices has yet to be comprehensively documented, particularly within the unique context of LMIC’s such as Mozambique. Our paper aimed to describe the perspectives of healthcare providers and people with DR-TB on the usability of simplified audiometry and ECG for monitoring treatment adverse effects.

## Methods

**Fig. 1:**
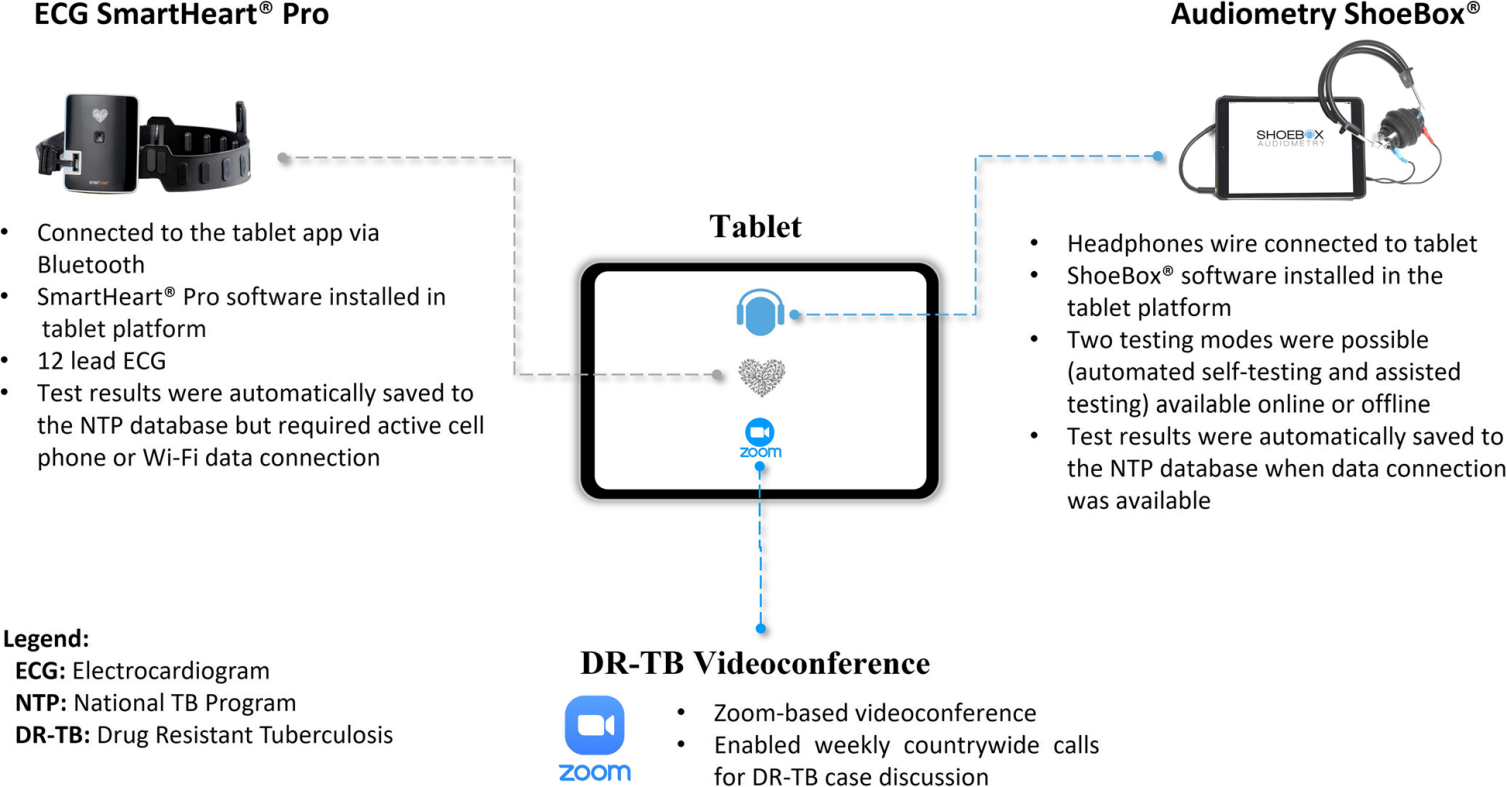
Integrated tablet platform

In December 2019, we conducted a qualitative study, using one-to-one in-depth interviews with front-line DR-TB healthcare providers who used the integrated tablet platform (Fig. 1). Simultaneously, Focus Group Discussions (FGD) were conducted with people with DR-TB who underwent assessments for treatment adverse events using the simplified devices at least once before the data collection initiation. The simplified devices were manufactured by ShoeBox (https://www.shoebox.md/), and Smartheart (https://www.smartheartpro.com/) companies.

### Study setting

Mozambique is a sub-Saharan and LMIC with an estimated 31 million inhabitants [12] and a high TB burden. The Mozambican National TB Program (NTP) reported 97,000 people with TB in 2021 and an estimated 3.7% of the new diagnosis and 20% of relapses are resistant to rifampicin (RR) or other drugs [10]. The country has a network of approximately 1700 primary health facilities. Of these, about 675 health facilities provided TB services (TB diagnosis and treatment) free of charge. In theory, all of them were also capable of caring for people with DR-TB. In practice, most people with DR-TB are treated in just 75 health facilities across the country and sixteen of them were chosen as the study’s implementation sites.

### Study sites

**Fig. 2:**
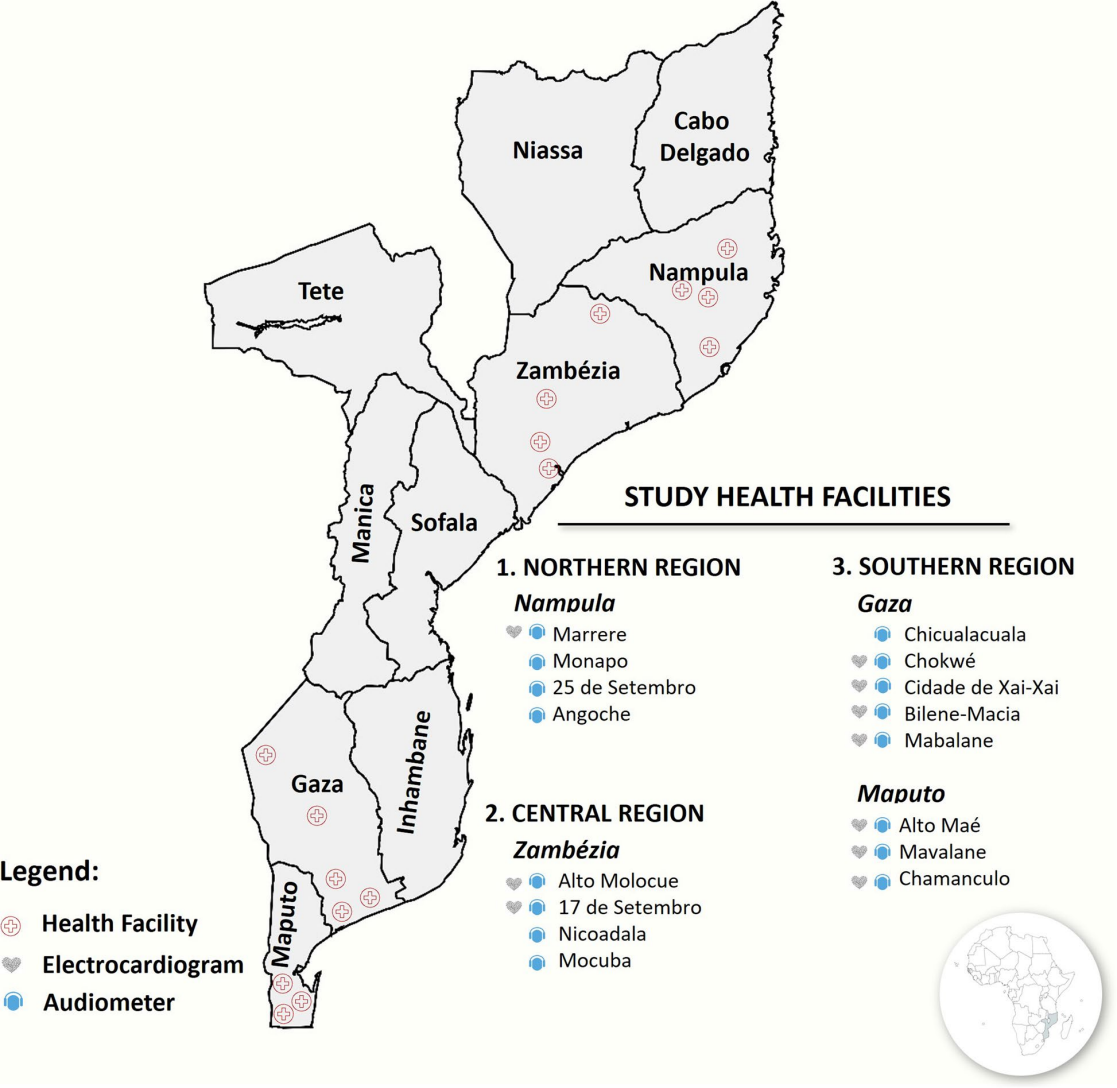
Map of Mozambique showing the study recruitment health facilities and the availability of an audiometer and EGC devices

The study was conducted in 16 outpatient health facilities equipped with the integrated tablets platform. Health facilities were selected to geographically represent the urban and peri-urban areas across provinces in the Northern, Central, and Southern regions of the country. Health facilities were eligible if they had the integrated tablet platform on-site for at least 6 months before the study initiation. All 16 health facilities had Audiometers in place and only 10 (62.5%) had ECG devices. Health facilities without an allocated ECG device referred people to the nearest health facility where the device was available. Provincial, central, and private hospitals were intentionally excluded due to non-representativeness of the primary care level and TB services. Since all provinces met the inclusion criteria, we randomly selected one province per region (Fig. 2). Additionally, we intentionally included Maputo City due to its peculiar characteristics, which encompass a densely populated environment, a high number of people with basic education, high level of technical expertise evident by a significant number of qualified staff, and better availability of resources and consumables.

### Study participants and data collection

#### Healthcare providers

Sixteen in-depth interviews were conducted with healthcare providers of different cadres, including health technicians, nurses, and doctors. Healthcare providers were purposively recruited and asked to participate. The Research Assistants (RA) obtained written informed consent, answered any questions about the study, and checked the eligibility criteria. Eligibility criteria were (i) being a healthcare provider exposed to DR-TB care, (ii) being exposed to the integrated tablet platform, (iii) agreeing to participate and provide written informed consent. The interviews focused on the perception of optimizing the safety of DR-TB treatment and included questions on the usability of simplified ECG, audiometry, and videoconference tools (see “In-depth interview guide” in Additional file 1). The interviews were conducted in Portuguese by AZ and PT and lasted between 26 to 64 min.

#### People with DR-TB

Fourteen FGD were conducted with People with DR-TB. The FGD’s study participants were conveniently recruited from an available list of people with DR-TB enrolled for care at primary care outpatients’ health facilities. The FDGs had 5 to 9 participants selected according to the following eligibility criterion: (i) 18 years of age or older, (ii) diagnosed with DR-TB and initiated on treatment, (iii) had been assessed, at least once, with either audiometer or ECG iv) agree to participate and to provide written informed consent. Participants were invited to participate in the study by their healthcare providers. Those who expressed interest were referred to the study team for recruitment, eligibility check, and consenting. The FGD guide primarily centered on participants’ perceptions of the practicality and usefulness of simplified testing devices. We specifically asked patients if they understood their disease and the importance of the audiometry and ECG exams during TB treatment (see “Focus group discussion interview guide” in Additional file 2). The FGDs were conducted in Portuguese and Chi-Changana in Maputo and Gaza, Portuguese and Emakua in Nampula, and Portuguese in Zambézia by AZ and PT with the support of a professional translator and lasted between 50 to 150 min. The final sample size, for both IDI and FGDs, was determined by data saturation defined as the point in the data collection process when new participants ceased to provide additional insights or perspectives related to the research question [13, 14].

### Analysis

All interviews were audio recorded and transcribed *verbatim* in Portuguese while study team members also took written notes for non-verbal communication. Categories and codes were created following the question guides and themes were generated to capture the underlying findings (see “Coding and analysis framework” in Additional file 3: Table S1). The transcripts were deductively coded by AZ, CN, PN, and RC in NVivo® software version 12, using a pre-defined codebook. Inductive coding was also conducted to account for emerging topics. Codes were compared for consistency and all discrepancies were resolved across the four investigators.

The analysis was carried out through a continuous interactive process involving data collection in the field, the transcription of digital recordings, and data coding to create a key summary for each batch of interviews. Trustworthiness was achieved by thoroughly reporting the methodology [15] and including quotes from the interview in the paper.

We generated two themes which are explained in detail below and fit our results to a conceptual framework consisting of three domains in the implementation of technological innovations in health [16]. The first domain addresses the safety concern unique to the implemented technology, the second addresses the optimization of the safe use of the implemented technology and discusses usability and acceptability (complete and correct use) of the implemented technologies and lastly, the third domain discusses the impact of the used technology in beneficiaries’ lives. Our study grounded the results and discussion in the second and third domains. This paper is reported according to the COREQ guidelines [17] (see the “COREQ checklist” in Additional file 4).

### Patient and public involvement

The TB coordinator and counselors at Health Facilities were involved in the identification of study participants. However, patients and the public were not involved in the design or planning of the study.

### Researcher’s characteristics and reflexivity

The research team consists of male and female researchers with diverse backgrounds and experience. The first author, PN, is a male medical doctor with 7 years of experience in qualitative research and 6 years of experience in clinical and epidemiological research in the field of TB. AZ is a male anthropologist with 5 years of experience in qualitative data collection and analysis. PT is a male anthropologist with experience in qualitative data collection. CN is a female sociologist with 2 years of experience in qualitative data collection and analysis and 5 years of experience with research in the TB field. Research assistants (RA) conducting data collection (AZ and PT) were naïve to the topic. They were trained on qualitative data collection techniques, study protocol, and topic guide by the first author (PN). They familiarised themselves with the topic guide and the context before commencing data collection. The RAs took notes of non-verbal aspects of the interview and shared a daily summary of the main findings with PN. Coding was conducted using original transcripts by AZ, CN, PN, and RC, who are all native Portuguese speakers and are proficient in Changana (AZ, VD, PN, and RC) and Emakua (RC). Excerpts from the transcripts and field notes were analyzed simultaneously.

## Results

### Socio-demographic characteristics

A total of 91 people with DR-TB were enrolled in the study. Of these, 21 (23%) were from Maputo City, 34 (37%) were from Gaza, 18 (20%) were from Nampula, and 18 (20%) were from Zambézia (Table 1). The overall median age in the four sites was 36 years (interquartile range [IQR] 27.0–45.0) and only 62.6% were assessed using ECG. All people were under a DR-TB long treatment regimen, which included injectable aminoglycoside drugs such as kanamycin and capreomycin. Most healthcare providers were male (13/16, 81%) and the overall median age was 33 years (IQR 29.8–35.5). Nearly half (9/16, 56%) were nurses and only one was a physician (1/16, 6%) (Table 2).

**Table 1:** Socio-demographic data of people with DR-TB per province. The time into DR-TB treatment was defined as the period from day 1 of treatment initiation until the day of the interview

	Maputo City *n* = *21*	Gaza *n* = *34*	Nampula *n* = *18*	Zambézia *n* = *18*	Total *n* = *91*
Median age (IQR)	36 (27.0–45.0)	39.5 (33.5–57.3)	33.5 (22.8–39.0)	30 (27.0–37.0)	36 (27.0–45.0)
Gender					
Male (%)	12 (57.1)	16 (47.1)	12 (66.7)	12 (66.7)	52 (57.1)
Marital status					
Married (%)	6 (28.6)	19 (55.9)	8 (44.5)	11 (61.1)	44 (48.4)
Never married/lived with someone (%)	12 (57.1)	15 (44.1)	5 (27.8)	6 (33.3)	38 (41.8)
Divorced (%)	2 (9.5)	0	4 (22.2)	0	6 (6.6)
Widow/widower (%)	1 (4.8)	0	1 (5.6)	1 (5.6)	3 (3.3)
Education					
Primary school (%)	6 (28.6)	16 (47.1)	9 (50)	5 (27.8)	36 (39.6)
Secondary school (%)	11 (52.4)	7 (20.6)	5 (27.8)	7 (38.9)	30 (33)
High school or professional degree (%)	1 (4.8)	2 (5.9)	1 (5.6)	5 (27.8)	9 (9.9)
Have not studied (%)	3 (14.3)	9 (26.5)	3 (16.7)	1 (5.6)	16 (17.6)
Occupation					
Currently employed/working (%)	17 (81)	24 (70.6)	11 (61.1)	10 (55.6)	62 (68.1)
Not currently employed/working (%)	4 (19.1)	10 (29.4)	7 (38.9)	8 (44.4)	29 (31.9)
Median time into DR-TB treatment in months (IQR)	9 (5.0–18.0)	7 (3.3–18)	6 (5.3–9.8)	11 (3.0–16.6)	7 (3.5–16.5)
People tested with the simplified devices					
Audiometer ShoeBox*®* (%)	21 (100)	33 (97.6)	18 (100)	18 (100)	90 (98.9)
ECG SmartHeart*®* Pro (%)	21 (100)	23 (67.7)	6 (33.3)	7 (30.8)	57 (62.6)

**Table 2:** Socio-demographic data of healthcare providers

	Maputo City *n* = *3*	Gaza *n* = *5*	Nampula *n* = *4*	Zambézia *n* = *4*	Total *n* = *16*
Median age (IQR)	30 (29.5–31)	37 (35–38)	31 (29.5–38.5)	31 (28.8–33.5)	32.5 (29.8–35.5)
Gender					
Male (%)	2 (66.7)	5 (100.0)	3 (75.0)	3 (75.0)	13 (81.3)
Female (%)	1 (33.3)	0 (0.0)	1 (25.0)	1 (25.0)	3 (18.8)
Healthcare worker cadre					
Physician (%)	0 (0.0)	1 (20.0)	0 (0.0)	0 (0.0)	1 (6.3)
Nurse (%)	2 (66.7)	3 (60.0)	2 (50.0)	2 (50.0)	9 (56.3)
Mid-level technicians (%)	1 (33.3)	1 (20.0)	2 (50.0)	2 (50.0)	6 (37.5)
Median work time in years (IQR)	6 (5.0–8.5)	11 (11.0–14.0)	6 (4.5–14.0)	4.5 (3.8–6.8)	6.5 (4.2–8.3)

### Theme 1: people with DR-TB had a limited understanding of the importance of Audiometry and ECG to their treatment

#### Knowledge of people with DR-TB about TB disease

Most people within the FGDs demonstrated a lack of understanding about DR-TB disease and treatment. Some people with DR-TB sought explanations based on their daily life knowledge or their personal experiences to explain what they meant by TB disease. A few participants related the disease to previous work in the mines, dust inhaled in the fieldwork, cigarettes, alcoholic beverages, sharing utensils, and feeding with someone infected and, very few were able to accurately explain what it was.

“Tuberculosis is a disease that the person catches in a windstorm or eats on the plate where the person [with TB] used it and did not wash it well, so the bacillus was not clean […]” (FGD, Patients, Mavalane health facility).

“[...] when you work in the mines, there are cases in which a lot of smoke comes out, so it creates tuberculosis” (FGD, Patients, Bilene health facility).

Some people with TB who were teachers associated TB with chalk dust inhaled during the teaching practice and others associated TB with sexual contact with a widow without complying with the kutchinga ceremony, a practice observed in many regions of Mozambique, that involves a ritualized sexual encounter between a widow and her brother-in-law (typically the deceased's younger brother) with the aim of cleansing or purifying the widow.

“I have been teaching since 2004, I have taught classes using chalk and now I am suffering from tuberculosis” (FGD, Patients, Bilene health facility).

“[…] before they said that tuberculosis is contracted through sexual intercourse, or by getting involved with a woman who had just lost her husband” (FGD, Patients, Bilene health facility).

#### Knowledge of people with DR-TB about the relevance of ECG and audiometry

In general, people with DR-TB understood the importance of audiometry and ECG to their treatment. They seemed to understand why audiometry and ECG exams were necessary assessments during the treatment, although these devices were recently placed in some health facilities across the country and the assessments were still new to TB services. Most people who contributed with consistent knowledge had at least a secondary school education and mostly attended care in an urban health facility.

“Audiometry is […] to control our hearing, for example, when I was at Machava [National TB Reference Hospital] I had a little pain and I couldn’t hear very well and with the device, they could assess my ability to hear” (FGD, Patients, Alto-Maé health facility).

#### Knowledge of healthcare providers about the relevance of ECG and audiometry

Healthcare providers seemed to have a solid understanding of the importance of both assessments for people with TB. Some reported a lack of regular training as a key factor that prevented the correct and complete use of these innovations. The limited number of allocated devices, mainly the ECGs, was mentioned as contributing to delaying or not performing the assessments, although there was a perceived effort to refer people to the nearest health facility to enable regular and complete testing.

“What I would like to see is more practice, learning interpretation of results and what should be done when we receive a result with clinical findings” (Male, Healthcare Provider, Marrere health facility).

### Theme 2: people with TB and healthcare providers attest to the usefulness of Audiometers and ECGs in the early detection of adverse drug effects

#### Perception of audiometer applicability in the diagnosis of adverse effects

There was a general perception that audiometry was of extreme importance as it allowed for quick detection of adverse effects and appropriate measures for the good of the people with DR-TB. Healthcare providers mentioned being able to perform audiology testing and reporting, however, the lack of human resources at health facilities was mentioned as an essential challenge.

#### Availability and usage of the ECG SmartHeart Pro

The ECG SmartHeart® was not widely distributed in the selected health facilities, despite the perceived utility of these devices. Only ten health facilities (out of 16) had ECG in place during data collection due to limitations in funding for this initiative. Those without an allocated ECG had to refer people to the nearest health facilities, which compromised the quality of the provided assessment. The ECG was reported to largely contribute to the quality of provided care enabling rapid diagnosis of adverse effects and adequate clinical measures to prevent the worsening of the disease state. It was also mentioned that some technicians, mainly those working at health facilities without an allocated device, had difficulties in adequately managing the devices, interpreting the results, and delivering reports to people.

#### Privacy and testing places within the health facilities

The surrounding noise and shared rooms hampered the correct and complete use of the devices. Interviews showed that the testing settings were not quiet and comfortable in many health facilities. The surrounding noise was found as an important factor influencing the quality of the audiometry while a lack of privacy in the ECG room resulted in people feeling uncomfortable as they had to uncover their bodies to perform the test.

“... It would be nice if we had a place where we could do the heart test and another place where we could have the hearing exam. Here when it’s midweek, it’s full of people and we take the exam in this place and people coming in and out can see our uncovered bodies...” (FGD, Patients, Mavalane health facility).

“... The space itself is very tight […] and it is close to the road. We are more likely to hear something else...” (FGD, Patients, Nicoadala health facility).

#### The use of the video conference system and complementary equipment for the innovations

Most healthcare providers mentioned that they had satisfactory knowledge about the system and its usage. Some healthcare providers pointed to mobile phone data network constraints when submitting clinical cases of TB to the national committee and attending weekly discussions.

The quality of the mobile phone data network was primarily mentioned in rural and remote health facilities. Other factors such as electricity supply, availability of printers, inks, and printing papers also played major roles. This was reported as relevant since, in some situations, people were requested to retake the exams because the quality of the first ones had been affected to some extent by one of these factors.

“For the audiometry, there are no challenges, but for the ECG, which is one of the devices that requires active internet communication (via the tablet’s data SIM card), and videoconferencing there have been some challenges with the network…” (Male, Healthcare provider, 17 de Setembro health facility).

## Discussion

Our study reports the experiences of people with DR-TB and healthcare providers regarding DR-TB treatment and the usability of simplified devices to monitor DR-TB treatment’s adverse effects. To the extent of our knowledge, this is the first manuscript to report such data in Mozambique.

Most people with DR-TB associated TB disease with their daily experiences, such as working in mines, exposure to dust, smoking, and alcohol consumption. Several studies have reported on the misconception about TB disease and transmission, resulting in inadequate adoption of preventive measures in the communities [18, 19]. These findings were also consistent with other studies in which people with TB attributed the disease to smoking, alcoholic habits, and other lifestyle habits [20]. A study in Georgia suggested that current and former people with TB had a good level of knowledge and awareness of the disease resulting from their experience of the treatment journey [21]. Our findings revealed that healthcare providers had a clear understanding of the disease, as they benefited from regular training and refreshments on the management of the disease, including screening, diagnosis, and treatment, as well as general knowledge of its transmission and prevention. This finding is consistent with other studies carried out elsewhere [22].

Our findings also suggested that people undergoing DR-TB treatment recognize the relevance of ECG and audiometry for the adequate monitoring of adverse effects, although these devices were recently placed in some health facilities nationwide. At the same time, healthcare providers showed a consolidated understanding of the usability and applicability of both devices to improve their work and provide better care for people. However, the lack of training in handling the ECG and interpreting the results compromised the correct and complete use of this innovation. Studies on digital health considered the lack of training as a barrier to the implementation of digital tools and argued that healthcare providers’ lack of training conditioned the quality of testing and reporting results [23, 24]. The simplified audiometry device was designed to be a self-testing tool; however, our study did not explore the challenges of people with TB using it themselves. Previous studies on digital health showed that there was great difficulty in implementing self-use innovations due to people’s lack of knowledge about the devices associated with their low level of education, which hampers better handling of different health innovations [25, 26].

Sharing of the room where the tests were performed and the surrounding noise was mentioned several times by healthcare providers and people with DR-TB as factors that compromised the quality of the audiometry and ECG tests [27–29]. A study by Storey, et al. [28] also reported the presence of surrounding noise at the audiometry testing place; however, the accuracy of the results was not affected by the noise. Furthermore, the lack of privacy in the ECG room created discomfort for people with DR-TB, who had to uncover their bodies to perform the test. Other factors such as the poor quality of the internet connection, interruptions of the electricity supply, printers, and availability of consumables, were described as barriers, as often people with DR-TB were asked to retake the exam due to failures on the first attempts. Similar findings were reported in a systematic review on mHealth where internet connection and electricity supply were found to be the main challenges for implementing health innovations in rural areas [30]. 

While there were challenges, with appropriate training and oversight, it is possible to use these platforms in LMIC and decentralized health facilities, assuming that internet connection is possible via a mobile phone network. Mozambique continues to use the Zoom platform to discuss complex DR-TB cases on a weekly basis, which we believe improves care, addresses adverse effects and, if appropriate, ensures that people with TB have access to recommended individualized drug regimens. With the recent pivot away from injectable aminoglycosides for the treatment of DR-TB, audiology screening is no longer regularly needed for people on all-oral regimens, and recent studies have also shown that very few people with DR-TB have clinically significant prolongation of the QTcF interval. Hearing loss is one of the main causes of morbidity in young children and adults, and routine screening and simple interventions using the ShoeBox® platform could address this public health issue. Heart disease is also a global health challenge, and access to high-quality ECGs at low cost would be a great benefit for the healthcare of people in LMIC. Countries will need to develop a strategy and a sustainable model to support these platforms in these and other contexts. Lastly, this study suggests some ways in which these platforms can be used successfully and challenges that would need to be addressed. However, more research is recommended into the correct, complete use and acceptability of the simplified devices, including challenges of implementing the innovations, especially in remote areas. In addition, validating the simplified audiometry and ECG in Mozambique is highly recommended. This sought to be nationally representative, including rural and urban health facilities in all regions, allowing for a better understanding of the phenomenon under investigation. To ensure the inclusion of all socioeconomic strata, most of the interviews with people with TB in Nampula province were conducted in Emakua (the most spoken language in Nampula). This required a translator who was proficient in both Portuguese and Emakua and who was also familiarised with the topic guides. The study team identified a qualified translator for this purpose. Although all the people with TB were under long treatment regimens with injectable aminoglycosides, data about specific treatment drugs and adverse effects were not collected. The literature on the usability of similar devices in health was limited, which prevented a better understanding of the phenomenon under study. It should be noted that recent WHO recommendations and Mozambican NTP guidelines have ended the use of injectable aminoglycosides in almost all DR-TB patients, in favor of shorter, exclusively oral regimens. As a result, the need for audiology testing has been eliminated for DR-TB. However, the NTP continues to hold weekly DR-TB clinical case discussions using the videoconferencing system and continues to carry out some ECG monitoring of adverse effects. Nonetheless, few clinically relevant adverse effects associated with ECG have been identified.

## Conclusions

Our study highlights the usage and understanding of the relevance of simplified tools for monitoring TB treatment adverse effects. Healthcare providers had a consolidated understanding of the importance of audiometry; however, the limited number of devices and lack of training hampered better service delivery. On the other hand, people with DR-TB perceived the value of these testing platforms as a quick and practical way to monitor the adverse effects of DR-TB treatment. Adequate training, network and electricity supply, and a variety of consumables were vital for the successful and correct use of these innovations.

## Supplementary Information

**Additional file 1.** In-depth interview guide.

**Additional file 2.** Focus group discussion guide.

**Additional file 3:** **Table S1.** Coding and analysis framework.

**Additional file 4.** COREQ checklist.

## Data Availability

The underlying data obtained in this study included audio recordings that cannot be shared as they contain identifying information. The interview guides coding and the framework are provided as Additional files 1, 2, and 3: Table S1.

## Abbreviations

BDQ: Bedaquiline
DLM: Delamanid
DR-TB: Drug Resistant Tuberculosis
ECG: Electrocardiogram
FGD: Focus Group Discussions
HAI: Health Alliance International
IDI: In-depth interviews
LMIC: Low- and middle-income countries
NTP: National TB Program
WHO: World Health Organization
