# Supplementary material for: Usability of simplified audiometry and electrocardiogram during treatment of drug-resistant tuberculosis in Mozambique: a qualitative study
Source: BMC Glob Public Health. 2024 Feb 14;2:12. doi: 10.1186/s44263-024-00039-4 (PMC11622995; doi:10.1186/s44263-024-00039-4)
Supplement: Supplementary file 1 — Additional file 1. In-depth interview guide. [file 44263_2024_39_MOESM1_ESM.pdf]

Health Alliance International (HAI)

Optimising the safety of Drug-Resistant Tuberculosis treatment with mobile audiometry and ECG testing for early detection of drug side effects

Semi-structured interview guide for data collection from health professionals providing care to people with DR-TB tested with audiometer ShoeBox and ECG SmartHeart Pro

TB-PS-AUD-ECG-ESE- I__I__I__I-I__I__I

Version 1.0 _ July 16, 2018

1. Instructions

1. Explain to the participant that:
   - This interview script aims to describe the perception of optimising the safety of Drug Resistant (DR) TB treatment using mobile audiometry and ECG testing for early detection of complications.
   - That he/she was invited to this interview because his contribution is essential to understanding the above-mentioned topics.
   - That this questionnaire will take about 30 to 45 minutes.
2. Inform them that if they agree to take part in the interview, we would like to record the interview so that we don't lose any important information they may give during the conversation. All the recorded information will be confidential, and the participant will not be identified by their names.
3. **Before we begin, we need to obtain informed consent and answer any questions the participant may have. The participant has the right not to answer any questions and to end the interview at any time.**

2. Date and place

Date I___I___I/I___I___I/I___I___I

Location (Province, District, Administrative Post, locality and Health Facility)

Province I___I___I___I___I___I___I___I___I___I___I___I___I

District I___I___I___I___I___I___I___I___I___I___I___I___I___I___I

Administrative Post I___I___I___I___I___I___I___I___I___I___I___I___I

Locality I___I___I___I___I___I___I___I___I___I___I___I___I___I___I___I

Health Facility I___I___I___I___I___I___I___I___I___I___I___I___I___I___I___I___I___I___I

3. Demographic characteristics

a) **What is your gender?**

Male I___I Female I___I Other I___I (please specify) ________________________________

b) **How old are you? I___I___I years**

c) **Please tell us your professional category** *(e.g. service assistant, nurse, medical doctor, health technician, etc.):*

Service assistant I___I Nurse I___I Medical Doctor I___I Health Technician I___I

Preventive Medicine Officer I___I Other (please specify) ____________________

Telephone number +I__I__I__I I__I__I I__I__I__I__I__I__I__I__I__I__I__I

4. General information from the semi-structured interview with health professionals

Ref. TB-AUD-ECG-ESE – I__I__I__I – I__I__I			
Date: I___I___I/ I___I___I/ I___I___I	Place: ____		
Initials of the participant I___I___I___I			
Start time I___I___I: I___I___I	End time I___I___I: I___I___I		
Language(s) Spoken ____			
**Results of the semi-structured interview**	Recorded ____ Not Recorded ____<br>Reasons for not recording ___________________________________<br>Interrupted ___ Not Interrupted ___<br>Reason for Interruption ____________________________________	Impossible to complete ____<br>To be completed on (Date) I___I___I / I___I___I / I___I___I	
**Facilitator Initials**: I__I__I__I	**Note taker Initials:** I__I__I__I		

5. Topic I (on drug-resistant tuberculosis)

	**Notes**
1. Can you explain what drug-resistant tuberculosis is?<br><br>[After the participant's answer, explain what drug-resistant tuberculosis is to avoid misunderstandings in the questions below]:<br><br>*[DR-TB is a tuberculosis infection caused by bacteria that are resistant to treatment with at least two of the strongest first-line anti-tuberculosis drugs, isoniazid and rifampicin*].<br><br>2. How is drug-resistant tuberculosis diagnosed in your health facility and the community?	

Explore:<br>*What is the purpose of diagnosing drug-resistant tuberculosis at your health facility and the community?*<br><br>*How do you identify people with drug-resistant tuberculosis in the community to diagnose them at the health facility?*<br><br>*What challenges do you face in diagnosing drug-resistant tuberculosis at your facility and in the community? And how do you overcome these challenges?*<br><br>*Are there other institutions in the community that diagnose drug-resistant tuberculosis? If* ***YES****, which ones? What is the purpose of this diagnosis?*<br><br>3. In your opinion, do people with DR-TB receive well the diagnosis? **IF NOT**, Why not?<br><br>***Explore:***<br>*Barriers and facilitators (for healthcare providers treating people with DR-TB).*<br><br>*Which age groups of people with DR-TB are the most cooperative and which are the most difficult to deal with?*<br><br>*Before this study, was it possible to do audiometry and ECG testing for People with DR-TB? How were the results used?*	

**6. Topic II (on the use of the audiometer and ECG in the Health Facility)**

1. What do you think about the process of implementing tablet-based audiometry and ECG testing?<br><br>2. How can the results of audiometry and ECG tests be useful for people with DR-TB and	**Notes**

why?<br><br>3. Do your health facilities use the SHOEBOX audiometer?<br><br>*If **YES**, can you please describe the flow that has been used to carry out consultation using ShoeBox? Can you share some challenges and successes with this platform? How was the process of testing and connecting to the internet? How can it be improved?*<br><br>4. In your health facilities, do you use the ECG SmartHeart Pro testing on people with DR-TB?<br><br>*If **YES**, can you please describe the flow that has been used to carry out consultation using this device? Can you share some challenges and successes with this platform? How was the process of testing and connecting to the internet? How can it be improved?*<br><br>5. What conditions exist in the health facilities for audiometry and ECG testing? Would you need any type of equipment, material, infrastructure, etc. that is not available now?<br><br>6. In technical terms, as a health professional at the health facilities, do you feel prepared to carry out audiometry and ECG testing? If **NOT,** why not? what would you need?<br><br>7. What was your experience of the acceptability of these tests by people with DR-TB? Was it possible for everyone to complete the tests and obtain valid results? If **NOT**, why not?<br><br>8. Is it worth continuing with this platform to perform audiometry and ECG tests on patients with DR-TB? How can it be improved?	

7. Topic III (on the use of the tablet for DR-TB ECHO videoconferencing)

1. What do you think about the process of implementing tablets to participate in DR-TB ECHO videoconferences?<br><br>2. Have you ever heard of DR-TB ECHO and videoconferencing?<br><br>*If **YES**, can you please describe how it works?*<br><br>3. In your facility, do you use a tablet-based or other system to participate in DR-TB ECHO and discuss clinical cases?<br><br>*If **YES**, can you please describe the flow that has been used to carry out these types of consultations? Can you share any challenges and successes with this platform? How was the process of testing, and the process of connecting to the internet? How can we improve? How many patients do you send to discuss clinical cases?*<br><br>4. What conditions exist in the health facilities for DR-TB ECHO videoconferencing?<br><br>5. In technical terms, do you feel prepared as a health professional at the health facilities to hold videoconferences? Why not? If **NOT**, what would you need?<br><br>6. Are you comfortable with the time of the videoconferences, which are usually held at 13:30 every Thursday? Would another time be better? **YES**, what time would be ideal?<br><br>7. Is it worth continuing to hold TB-MR ECHO videoconferences? Why not? If **YES** or **NO**, how can we improve?	**Notes**

1. Observations

END OF THE INTERVIEW
