# Supplementary material for: Usability of simplified audiometry and electrocardiogram during treatment of drug-resistant tuberculosis in Mozambique: a qualitative study
Source: BMC Glob Public Health. 2024 Feb 14;2:12. doi: 10.1186/s44263-024-00039-4 (PMC11622995; doi:10.1186/s44263-024-00039-4)
Supplement: Supplementary file 2 — Additional file 2. Focus group discussion guide. [file 44263_2024_39_MOESM2_ESM.pdf]

Health Alliance International (HAI)

Optimising the safety of Drug-Resistant Tuberculosis treatment with mobile audiometry and ECG tests for early detection of drug side effects

Focus Group Discussion with Patients Tested with ShoeBox Audiometer and ECG SmartHeart Pro

TB-PT-AUD-ECG-DGF- I__I__I__I – I__I__I

Version 1.0_16 July 2018

1. INSTRUCTIONS

1. Explain to the participants that:

- This focus group discussion aims to gain insight into optimising the safety of Drug-Resistant (DR) Tuberculosis with mobile audiometry and ECG testing for early detection of complications.
- You were invited to this interview because their contribution is essential to understanding how mobile audiometry and ECG help the diagnosis of drug side effects.
- That the focus group discussion will take around 1 to 1.30 hours.

2. Discuss with them that, if they allow it, the focus group discussion will be recorded, to avoid losing the important information they give during the conversation. All the information recorded will be confidential and the participants will not be identified by their names. Still, as it is a conversation between several people, we cannot guarantee that the other participants will not reveal information that has been said in this group. If you don't agree to the discussion being recorded, one of the interviewers will take notes while the conversation takes place.

3. Before starting we need to obtain informed consent and answer any questions the participant may have. The participant has the right not to answer any questions, and to end the interview at any time.

2. DEMOGRAPHIC INFORMATION OF PARTICIPANTS

	**Participant 1**	**Participant 2**	**Participant 3**	**Participant 4**	**Participant 5**	**Participant 6**	**Participant 7**
**Gender**	□ Male. (1)<br>□ Female. (2)	□ Male. (1)<br>□ Female. (2)	□ Male. (1)<br>□ Female. (2)	□ Male. (1)<br>□ Female. (2)	□ Male. (1)<br>□ Female. (2)	□ Male. (1)<br>□ Female. (2)	□ Male. (1)<br>□ Female. (2)
**Person with DR-TB who has already been tested with devices**	□ Shoebox Audiometer<br>□ ECG Smartheart Pro	□ Shoebox Audiometer<br>□ ECG Smartheart Pro	□ Shoebox Audiometer<br>□ ECG Smartheart Pro	□ Shoebox Audiometer<br>□ ECG Smartheart Pro	□ Shoebox Audiometer<br>□ ECG Smartheart Pro	□ Shoebox Audiometer<br>□ ECG Smartheart Pro	□ Shoebox Audiometer<br>□ ECG Smartheart Pro
**Time into DR-TB treatment**<br><br>**Age**	Months: ______<br><br>Years: ______	Months: ______<br><br>Years: ______	Months: ______<br><br>Years: ______	Months: ______<br><br>Years: ______	Months: ______<br><br>Years: ______	Months: ______<br><br>Years: ______	Months: ______<br><br>Years: ______
**Marital Status**<br><br>**Religion**<br><br>**Occupation**<br><br>**Level of education**<br><br>**How many years in total did you study?**	□ Single<br>□ Married<br>□ Widow<br>□ Other<br>Specify: ___________<br><br>_____________<br><br>_____________<br><br>□ Primary<br>□ Secondary<br>□ University<br>□ Other<br>Specify: ___________<br><br>Years: ______	□ Single<br>□ Married<br>□ Widow<br>□ Other<br>Specify: ___________<br><br>_____________<br><br>_____________<br><br>□ Primary<br>□ Secondary<br>□ University<br>□ Other<br>Specify: ___________<br><br>Years: ______	□ Single<br>□ Married<br>□ Widow<br>□ Other<br>Specify: ___________<br><br>_____________<br><br>_____________<br><br>□ Primary<br>□ Secondary<br>□ University<br>□ Other<br>Specify: ___________<br><br>Years: ______	□ Single<br>□ Married<br>□ Widow<br>□ Other<br>Specify: ___________<br><br>_____________<br><br>_____________<br><br>□ Primary<br>□ Secondary<br>□ University<br>□ Other<br>Specify: ___________<br><br>Years: ______	□ Single<br>□ Married<br>□ Widow<br>□ Other<br>Specify: ___________<br><br>_____________<br><br>_____________<br><br>□ Primary<br>□ Secondary<br>□ University<br>□ Other<br>Specify: ___________<br><br>Years: ______	□ Single<br>□ Married<br>□ Widow<br>□ Other<br>Specify: ___________<br><br>_____________<br><br>_____________<br><br>□ Primary<br>□ Secondary<br>□ University<br>□ Other<br>Specify: ___________<br><br>Years: ______	□ Single<br>□ Married<br>□ Widow<br>□ Other<br>Specify: ___________<br><br>_____________<br><br>_____________<br><br>□ Primary<br>□ Secondary<br>□ University<br>□ Other<br>Specify: ___________<br><br>Years: ______

	**Participant 8**	**Participant 9**	**Participant 10**	**Participant 11**	**Participant 12**
**Gender**	□ Male. (1)<br>□ Female. (2)	□ Male. (1)<br>□ Female. (2)	□ Male. (1)<br>□ Female. (2)	□ Male. (1)<br>□ Female. (2)	□ Male. (1)<br>□ Female. (2)
**Person with DR-TB who has already been tested with devices**	□ Shoebox Audiometer<br>□ ECG Smartheart Pro	□ Shoebox Audiometer<br>□ ECG Smartheart Pro	□ Shoebox Audiometer<br>□ ECG Smartheart Pro	□ Shoebox Audiometer<br>□ ECG Smartheart Pro	□ Shoebox Audiometer<br>□ ECG Smartheart Pro
**Time into DR-TB treatment**<br><br>**Age**	Meses: ______<br><br>Anos: ______	Meses: ______<br><br>Anos: ______	Meses: ______<br><br>Anos: ______	Meses: ______<br><br>Anos: ______	Meses: ______<br><br>Anos: ______
**Marital Status**<br><br>**Religion**	□ Single<br>□ Married<br>□ Widow<br>□ Other<br>Specify: ___________<br><br>_____________	□ Single<br>□ Married<br>□ Widow<br>□ Other<br>Specify: ___________<br><br>_____________	□ Single<br>□ Married<br>□ Widow<br>□ Other<br>Specify: ___________<br><br>_____________	□ Single<br>□ Married<br>□ Widow<br>□ Other<br>Specify: ___________<br><br>_____________	□ Single<br>□ Married<br>□ Widow<br>□ Other<br>Specify: ___________<br><br>_____________

**Occupation**	___________	___________	___________	___________	___________
**Level of education**	□ Primary<br>□ Secondary<br>□ University<br>□ Other<br>Specify:<br>__________	□ Primary<br>□ Secondary<br>□ University<br>□ Other<br>Specify:<br>__________	□ Primary<br>□ Secondary<br>□ University<br>□ Other<br>Specify:<br>__________	□ Primary<br>□ Secondary<br>□ University<br>□ Other<br>Specify:<br>__________	□ Primary<br>□ Secondary<br>□ University<br>□ Other<br>Specify:<br>__________
**How many years in total did you study?**	Years: ______	Years: ______	Years: ______	Years: ______	Years: ______

3. GENERAL INFORMATION FROM THE FOCUS GROUP DISCUSSION

Ref. TB-AUD-ECG-DGF – I__I__I__I – I__I__I		
Date: I___I___I/ I___I___I/ I___I___I	Place: ____	
Initial number of participants: I__I__I	Final number of participants: I__I__I	
Start time: I__I__I: I__I__I	End time: I__I__I: I__I__I	
Interview Language(s)<br>____		
**Results of the focus group discussion**	Recorded ____ Not Recorded ____<br>Reasons for not recording<br>__________________________________<br>Interrupted ___ Not Interrupted ___<br>Reason for Interruption<br>___________________________________	Impossible to complete ____<br>To be completed on (Date)<br>I___I___I / I___I___I / I___I___I
**Facilitator** I__I__I__I<br>Facilitator's initials	**Note taker** I__I__I__I<br>Note taker's initials	

4. Topic I: About drug-resistant tuberculosis

1. Please explain what tuberculosis is.<br><br>**2.** Please explain what drug-resistant tuberculosis is?<br><br>[After the participants have answered, explain what drug-resistant tuberculosis is to avoid misunderstandings in the questions below]:<br><br>*[MDR-TB is a tuberculosis infection caused by bacteria that are resistant to treatment with at least two of the strongest first-line anti-tuberculosis drugs, isoniazid and rifampicin]*<br><br>3. How were you diagnosed with tuberculosis when you fell ill?<br>4. How did you react when you were diagnosed with MDR-TB?<br>5. What is the importance of diagnosing MDR-TB in the community?<br>6. Can you comment on the main challenges you have had in the community and in the health facility in achieving DR-TB treatment?	**Notes**

5. TEMA II: Sobre o audiómetro e ECG

7. Have you ever heard about audiometry or ECG? Have you attended or been to a consultation where an audiometer or ECG was used?<br>a) **If YES, what was your experience of these tests?**<br><br>**Explore:** i) how was it carried out? ii) What is the use of the audiometer and ECG?<br><br>After the participant's answer:<br><br>*[Explain what audiometer and ECG are in the context of the project and how they are carried out, to avoid misunderstandings in the questions below: in the context of the project, Audiometer is a an equipment used to carry out the Audiometry test, which assesses a patient's ability to hear sounds. Injectable drugs for DR-TB treatment can cause ototoxicity, so it is recommended that everyone has a routine audiometry test during treatment with injectables].*	**Notes**

[ECG is a test that allows the heart rhythm to be recorded. The ECG consists of precisely studying the heart's activity using electrodes placed on the patient's chest, wrist and ankle. Cardiac activity is measured at various points in the heart, called leads, and is recorded in the form of a curve for each lead. Some DR-TB drugs can cause changes in the rhythm that we need to monitor].<br><br>Explain that we will first talk about the possibility of doing audiometry and ECG in the health unit.<br><br>8. What do you think about audiometry and ECG tests being carried out at the health unit?<br><br>9. Have you ever done a self-audiometry testing using a tablet? If YES, can you tell us about your experiences with the test?<br><br>**Explore:** i) What was successful or difficult and why? ii)? What was good? iii) What was the challenge? iv) How do you think the process could be improved?<br><br>10. What conditions exist in the health facility for audiometry and ECG tests?<br><br>**Explore:** i) the place where the tests are carried out. ii) the environment.<br><br>12. In your opinion, which health professionals should carry out the audiometry and ECG tests?<br><br>**Explore:** i) With whom they feel most comfortable (gender, age, professional category)	

6. Observations

____

____

____

____

____

____

____

____

____

____

____

____

____

____

END OF INTERVIEW
