# Supplementary material for: Usability of simplified audiometry and electrocardiogram during treatment of drug-resistant tuberculosis in Mozambique: a qualitative study
Source: BMC Glob Public Health. 2024 Feb 14;2:12. doi: 10.1186/s44263-024-00039-4 (PMC11622995; doi:10.1186/s44263-024-00039-4)
Supplement: Supplementary file 3 — Additional file 3: Table S1. Coding and analysis framework. [file 44263_2024_39_MOESM3_ESM.pdf]

Additional file 3: Table. S1: Coding and analysis framework

Pre-analysis (Coding)		Analysis	
**Sub-codes**	**Codes**	**Category**	**Themes**
Perspectives of people with DR-TB	Knowledge about TB and DR-TB	Understanding of people with DR-TB about TB disease	People with DR-TB had a limited understanding of the importance of Audiometry and ECG to their treatment
Perspectives of healthcare providers			
Perspectives of people with DR-TB	Knowledge about ECG and Audiometry	Understanding of people with DR-TB about the relevance of ECG and Audiometry	
Perspectives of healthcare providers	Knowledge about ECG and Audiometry	Understanding of healthcare providers about the relevance of ECG and Audiometry	
Performing the test	Usage of ShoeBox® Audiometers	Perception of Audiometer applicability in the diagnosis of adverse effects	**People with TB and healthcare providers attest to the usefulness of Audiometers and ECGs in the early detection of adverse drug effects**
Relevance of results for patients			
Equipment and complementary materials			
Equipment availability	Usage of Audiometers ECG SmartHeart® Pro	Availability and usage of the ECG SmartHeart Pro	
Performing the test			
Equipment and complementary materials			
–	Performing the test	Privacy and testing place at the health facilities	
Weekly case discussion	Video conference system	Use of the video conference system and complementary equipment for the innovations	
